# Supplementary material for: Compulsive alcohol drinking in rodents is associated with altered representations of behavioral control and seeking in dorsal medial prefrontal cortex
Source: Nat Commun. 2022 Jul 9;13:3990. doi: 10.1038/s41467-022-31731-4 (PMC9271071; doi:10.1038/s41467-022-31731-4)
Supplement: Supplementary file 1 — Supplementary Information [file 41467_2022_31731_MOESM1_ESM.pdf]

Supplemental

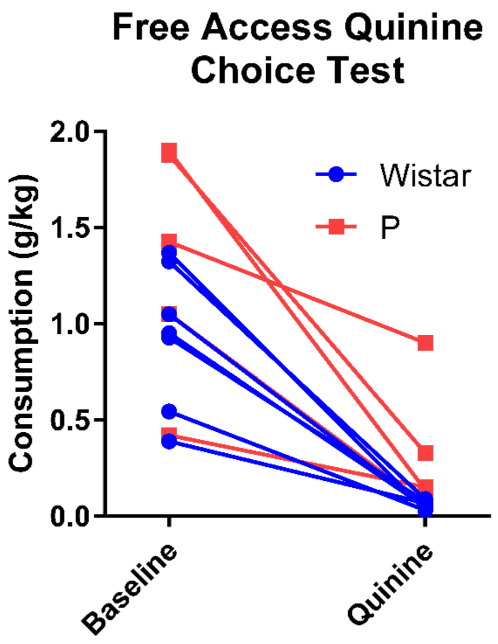

Supplemental Figure 1: Free access quinine choice test. Animals were given approximately 60 minutes of free access to both sippers filled with 10% ethanol on baseline days (total time matched to regular 2CAP session, sippers extended into the chamber throughout the whole session). During a subsequent session, the preferred sipper from the baseline session was adulterated with 0.1 g/L quinine and the non-preferred sipper contained 10% ethanol as usual. During this quinine session, the animal was manually required to sample both sippers twice. Two P rats were not included. One was lost prior to this test. For the other, the non-preferred sipper was accidentally adulterated with quinine. All animals demonstrated a preference for regular alcohol over alcohol adulterated with 0.1 g/L quinine (strain main effect: $F(1,20)=4.499$, $p=0.0466$, liquid main effect: $F(1,20)=37.4$, $p<10^{-4}$, interaction: $F(1,20)=0.20$, $p=0.66$; paired t-test, P rats: $t(4)=3.56$, $p=0.024$, Wistars: $t(6)=6.38$, $p=0.000698$).

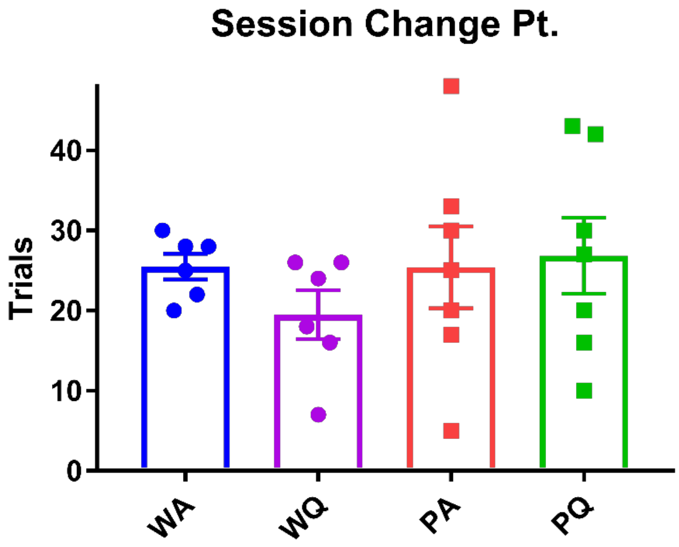

Supplemental Figure 2: Session change point was not affected by animal strain or liquid type. (N=6 Wistars, N=7 P rats. Data are presented as mean +/- sem.)

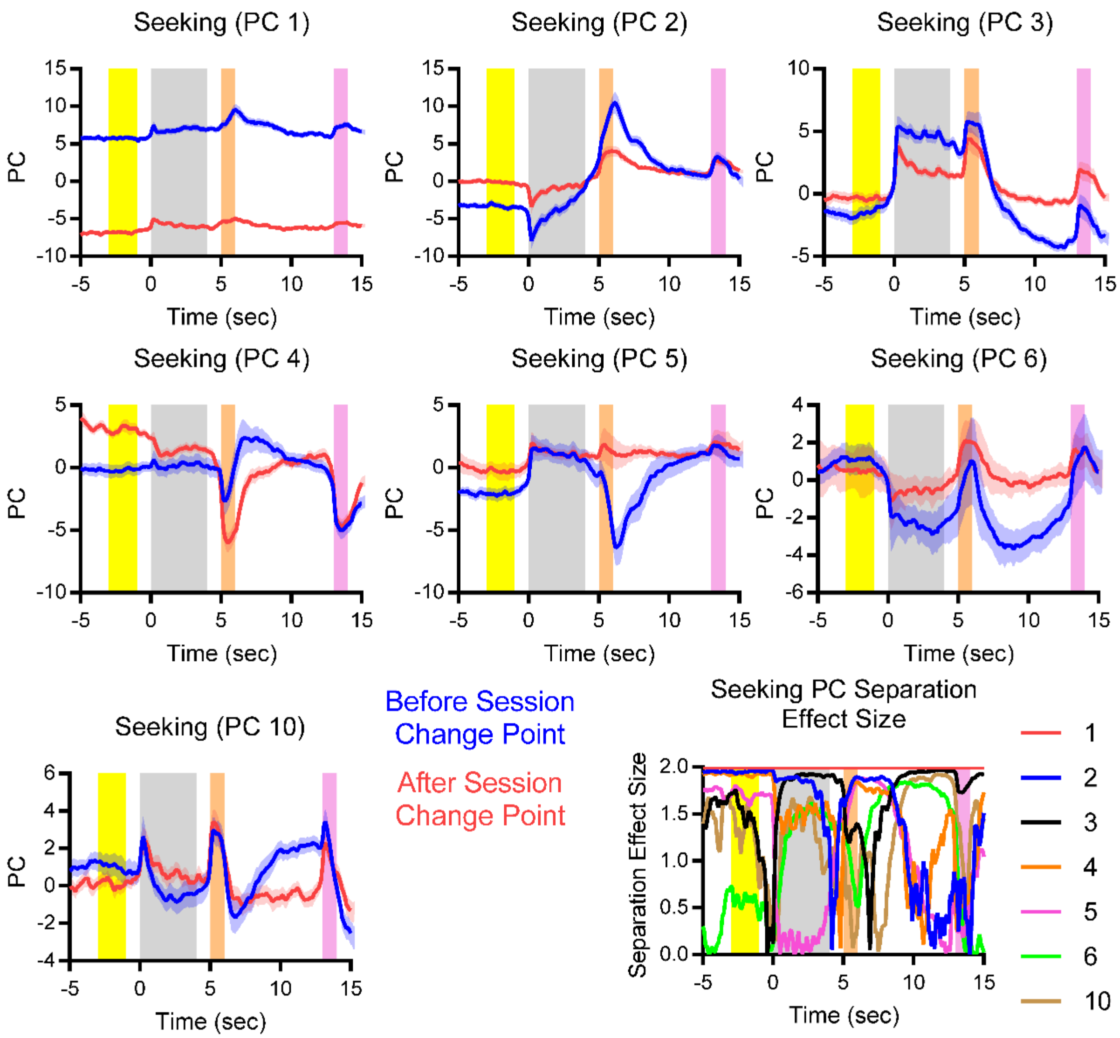

Supplemental Figure 3: All stable PCs (all neurons) for trials before and after session change point (mean +/- std across PCA subsample trials). Recall, approaching decreased substantially following session change point, marking a change in seeking state. Seeking representation was assessed during the yellow epoch prior to CS+ onset. Many PCs (especially 1, 2, and 4) showed robust separation during the seeking epoch.

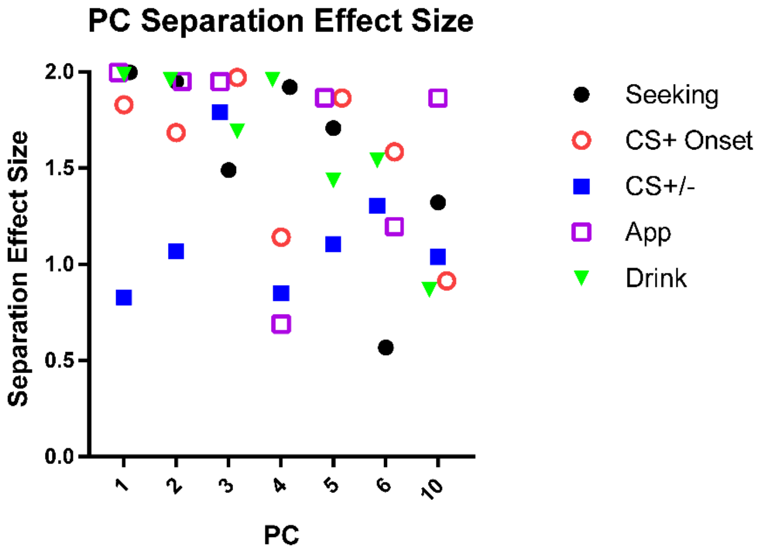

Supplemental Figure 4: PC separation effect sizes across epochs of interest for each type of representation. Effect size was calculated using Cohen's d using individual PCA subsampling trials and the epochs of interest.

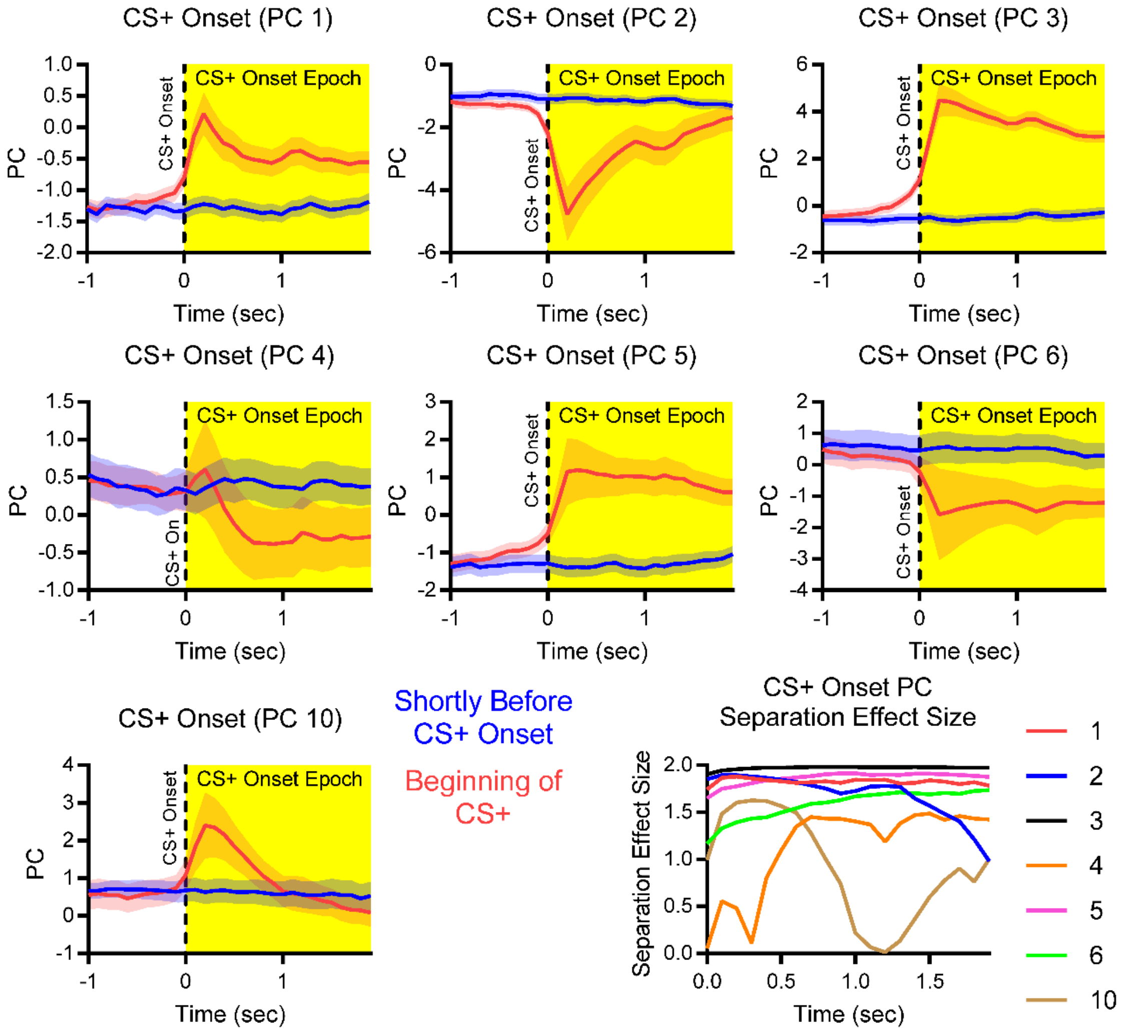

Supplemental Figure 5: All stable PCs (all neurons) averaged over 2 seconds shortly before CS+ onset (-2.4 to -0.5 seconds before CS+ onset) and during the first 2 seconds of the CS+ (0 to 1.9 seconds after CS+ onset) (mean +/- std across PCA subsample trials). CS+ onset representations were assessed by comparing these epochs, though more time shown for clarity. Many PCs (especially 3 and 5) showed robust separation during the CS+ onset epoch.

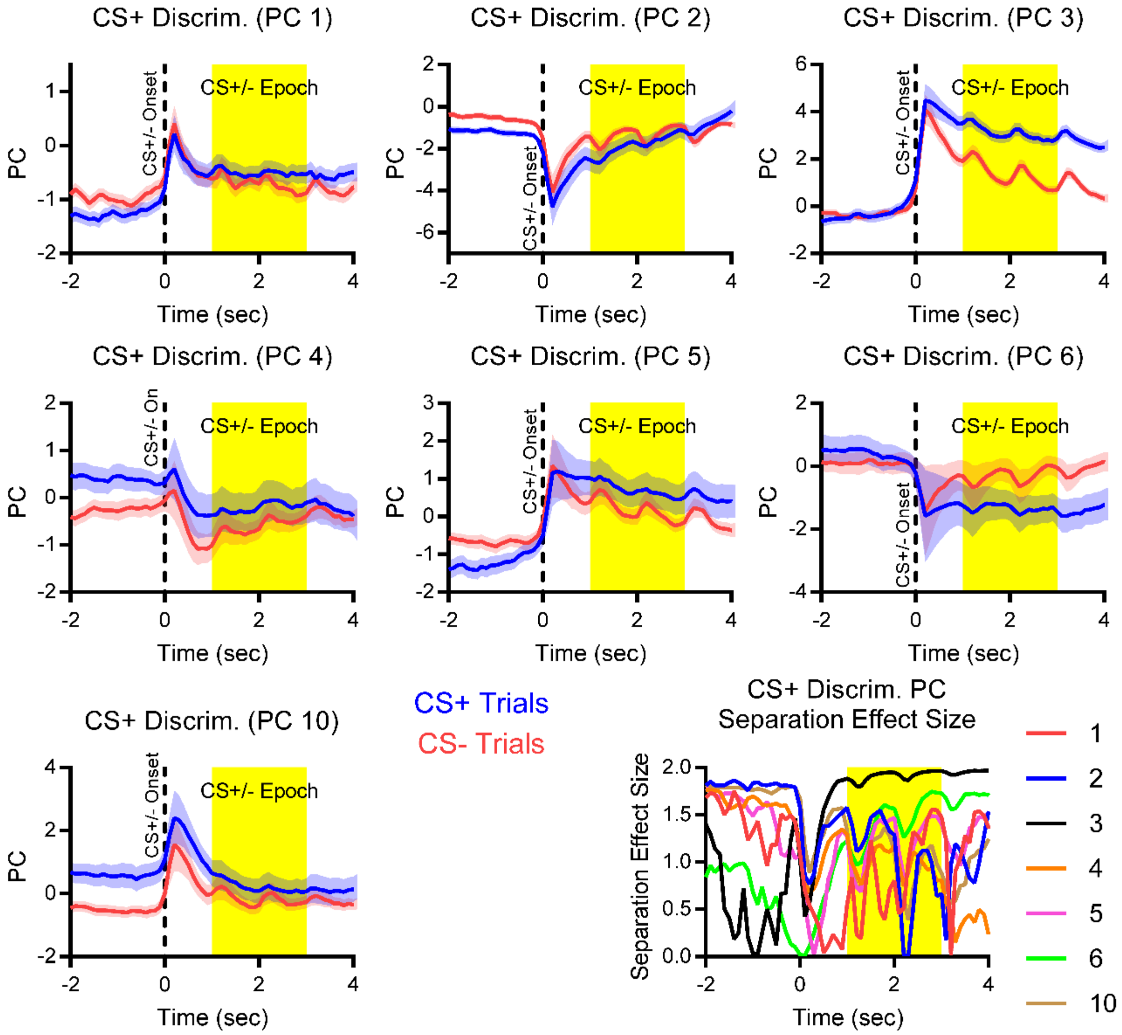

Supplemental Figure 6: All stable PCs (all neurons) averaged over CS+ and CS- trials (mean +/- std across PCA subsample trials). CS+ vs. CS- representation strength was assessed by comparing the CS+ and CS- PC values during the CS+/- epoch (yellow region). PC separation was weakest for CS discrimination, with PCs 3 and 6 showing the largest separation.

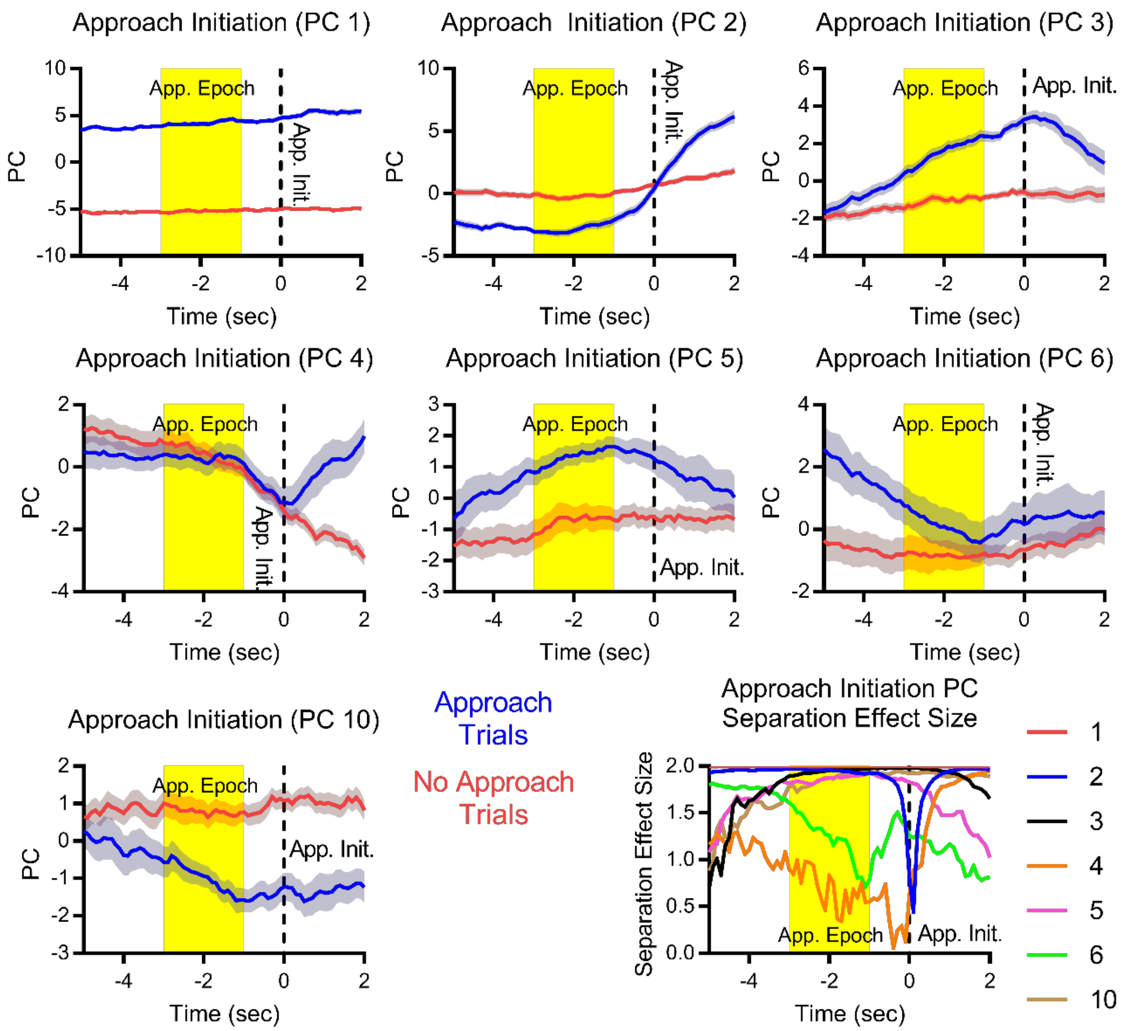

Supplemental Figure 7: All stable PCs (all neurons) averaged over approach and no approach trials time locked to the approach initiation time point found using the change point in the within trial likelihood to approach (mean +/- std across PCA subsample trials). Approach initiation representation strength was assessed by comparing PC trajectories on approach and no approach trials during the approach initiation time window (yellow epoch). Most PCs showed robust separation during the approach initiation epoch, many with distinct dynamics over the time window shown.

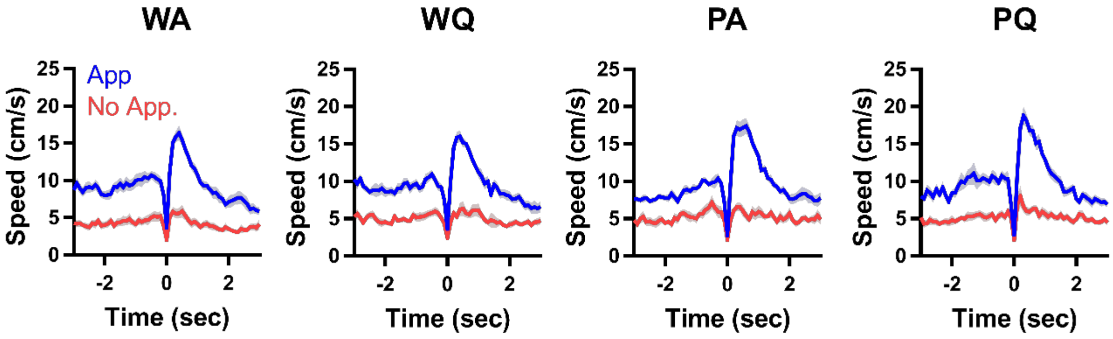

Supplemental Figure 8: Speed profiles time locked to approach initiation for all sessions. Approach trials tended to have higher speed before approach initiation and a large increase in speed following approach initiation. W: Wistar. P: P rat. A: alcohol. Q: quinine. Mean +/- sem.

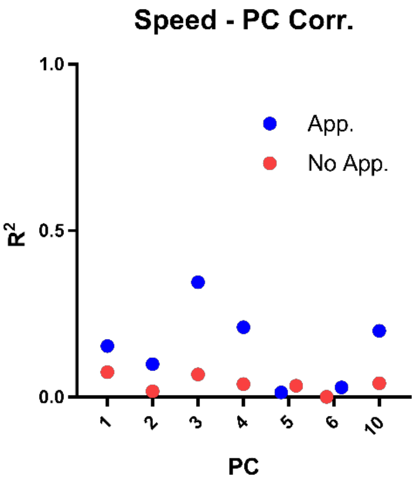

Supplemental Figure 9: PC scores were weakly correlated with animal speed. From 3 seconds before to 2 seconds after approach initiation, the correlation between the speed (Supplemental Figure 8) and the PC scores (Supplemental Figure 7) was low. Furthermore, no PCs exhibited sharp changes near the approach initiation, despite the presence of characteristic decreases in speed near approach initiation. This indicates that these PCs were not simply movement artifacts or represented only movement.

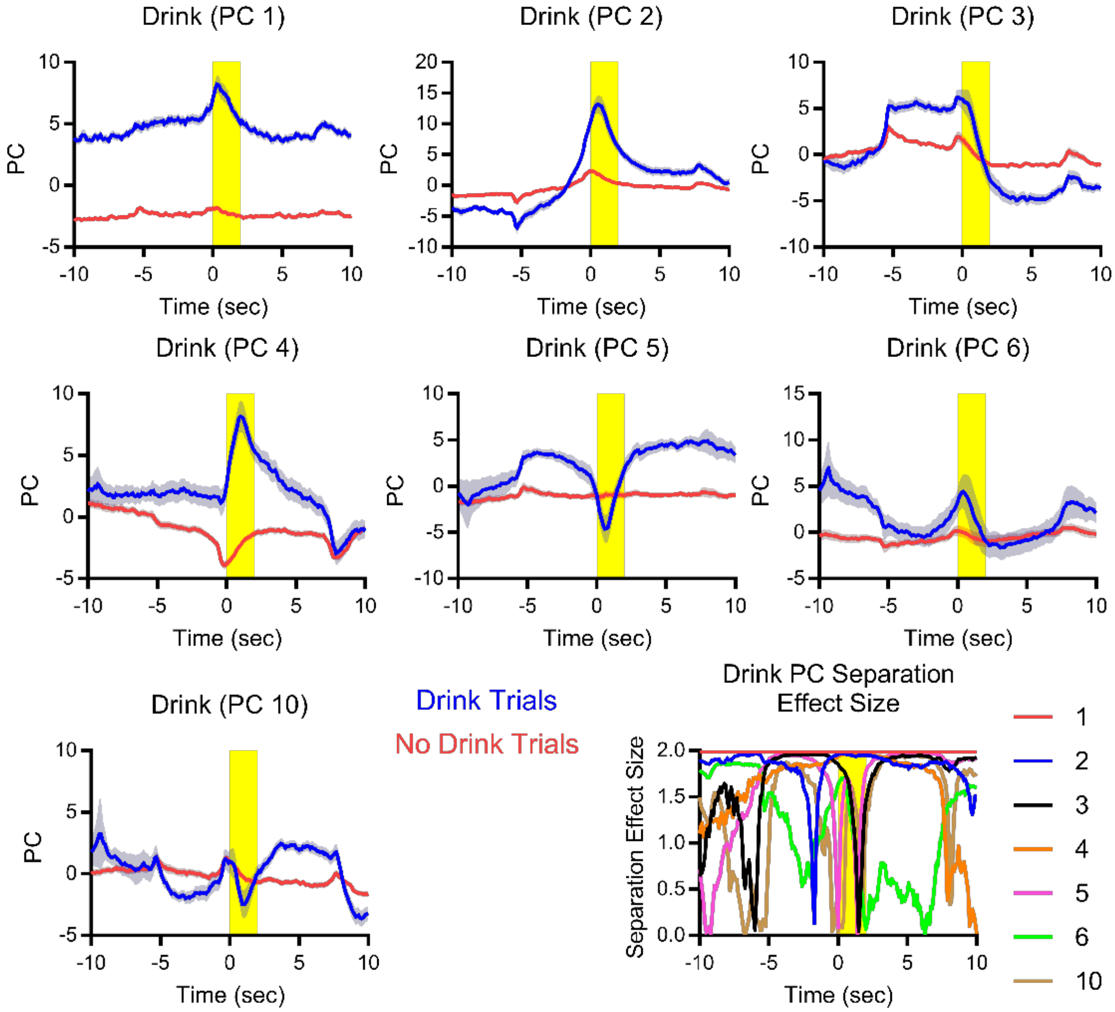

Supplemental Figure 10: All stable PCs (all neurons used) averaged over drink and no drink trials time locked to the time the animal arrived at the sipper (mean +/- std across PCA subsample trials). Drink encoding was assessed by comparing PC trajectories on drink and no drink trials during the drink time window (yellow epoch). Many PCs showed robust separation during the drink epoch, many with distinct dynamics over the time window shown.

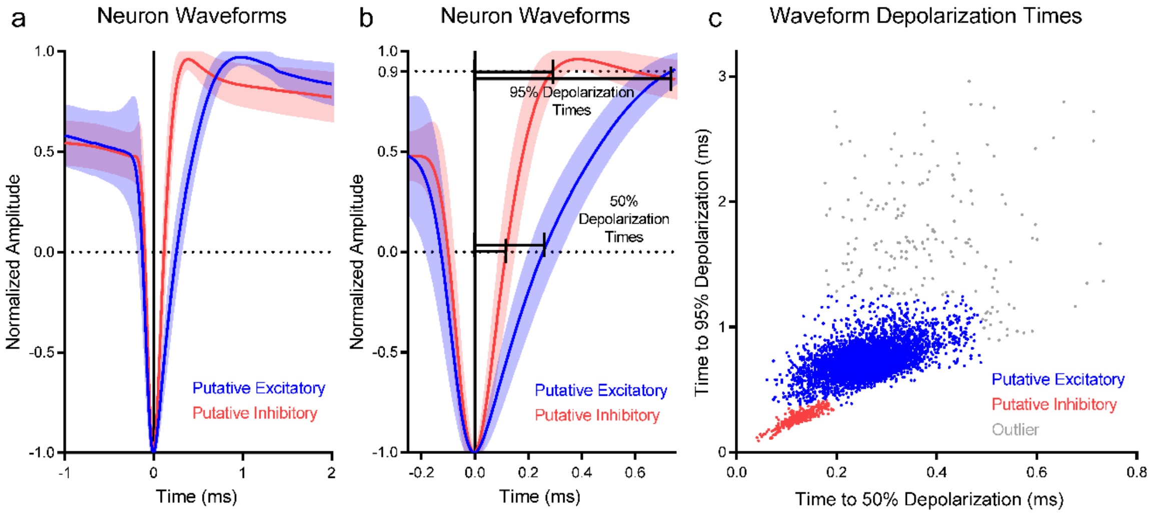

Supplemental Figure 11: Putative excitatory and inhibitory neuron classification by waveform. a, Average neuron waveforms (taken from electrode with largest amplitude) were scaled to range from -1 to 1. **b**, To measure how fast the action potential depolarized, we assessed the time it took the average waveform to reach 50% (0) and 95% (0.9) of the maximum depolarization. **c**, When these times were plotted, two clusters and outliers were apparent. Manual cluster was performed to label neurons as putative excitatory, putative inhibitory, or outlier (excluded from firing rate response analysis). a-b: mean +/- std.

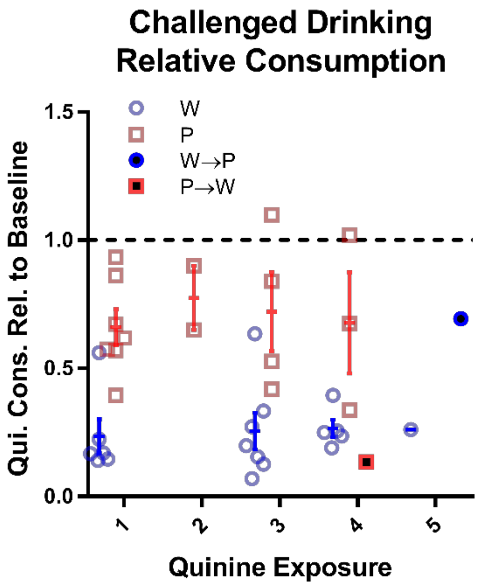

Supplemental Figure 12: Challenged drinking behavior was largely consistent across later sessions. Relative consumption between challenged drinking (quinine) and baseline consumption on the previous day remained relatively unchanged across additional quinine exposures. Sample sizes varied across quinine exposures due to ordering of experimental sessions. For all Wistars, the second quinine exposure was the free access quinine test. One Wistar and one P rat were switched between groups for analysis of

electrophysiology (see Supplemental Fig. 14). (N=7 Wistars. N=7 P rats. Data are presented as mean +/- sem.)

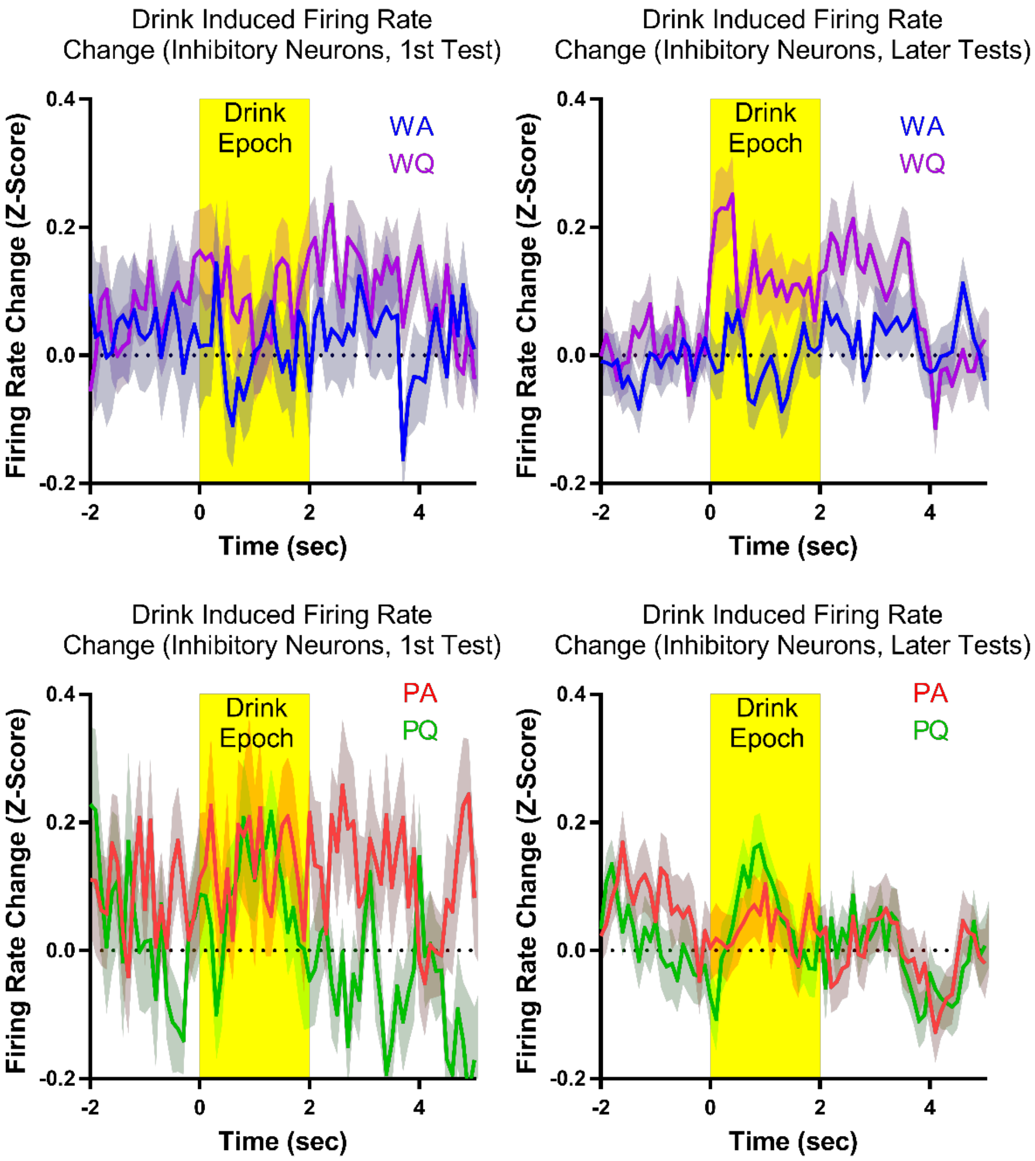

Supplemental Figure 13: Inhibitory neuron firing rate responses to drink trials. Significant differences were found for the mean firing rate change during the drink epoch in Wistars during later compulsive drinking tests. Mean +/- sem change in firing rate on drink trials, see Figure 7 c and f for number of neurons.

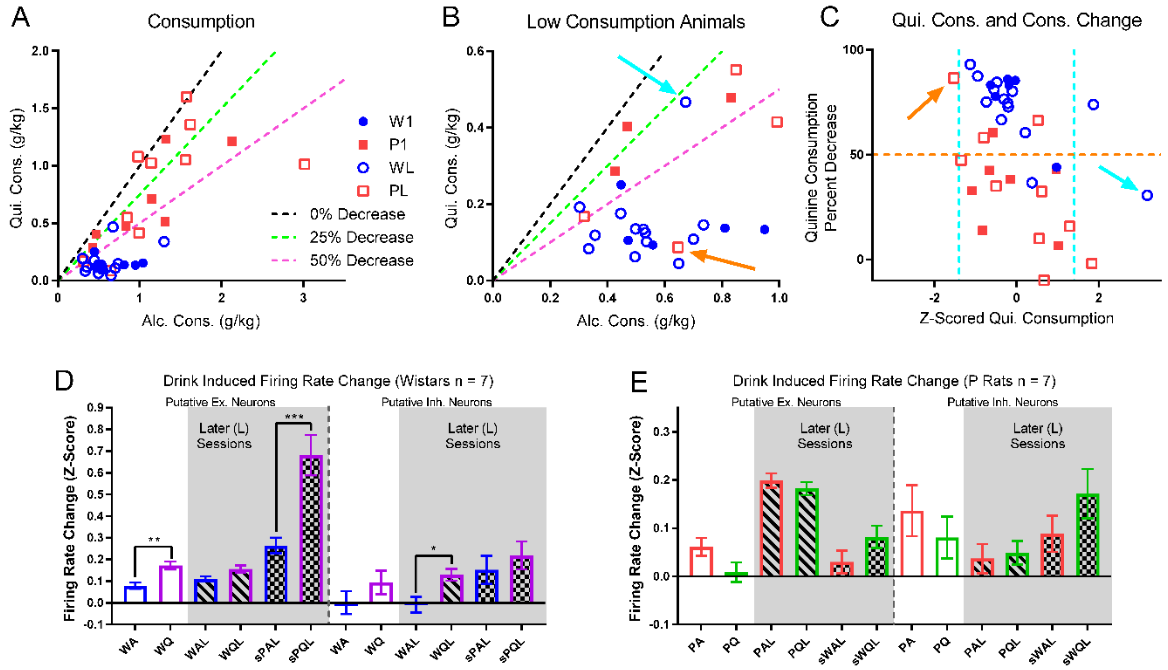

Supplemental Figure 14: For later recordings, in the PCA, two recordings were switched between groups based on alcohol+quinine consumption (**a-c**). One Wistar recording had high alcohol+quinine consumption, exhibited a small decrease in consumption during the alcohol+quinine session, and was therefore reclassified as a compulsive session (cyan arrow). One P rat recording had low alcohol+quinine consumption, exhibited a large decrease in consumption during the alcohol+quinine session, and was therefore reclassified as a non-compulsive session (orange arrow). W1: Wistars, first compulsive drinking test. P1: P rats, first compulsive drinking test. WL: Wistars, later compulsive drinking tests. PL: P rats, later compulsive drinking tests. **d**, Comparison of neuron firing rate responses during drinking between all non-compulsive subjects (WA, WQ, WAL, WQL) (Wistars and the one switched P rat test sequence) to the firing rate responses for just the switched P rat test sequence (sPAL and sPQL). The switched P rat compulsive test sequence recordings demonstrated a significant increase in putative excitatory neuron firing (post-hoc Tukey's test: $p=2.7*10^{-5}$) during quinine adulteration. **e**, Comparison of neuron firing rate responses during drinking between all compulsive subjects (PA, PQ, PAL, PQL) (P rats and the one switched Wistar test sequence) to the firing rate responses for just the switched Wistar test sequence (sWAL and sWQL). The switched Wistar test sequence demonstrated no significant differences in putative excitatory or inhibitory neuron firing during quinine adulteration. These results were similar to those observed for all non-compulsive and compulsive subjects, which increases confidence in switching these compulsive test data between groups. See Methods section for more details. (In d and e, data are presented as mean +/- sem. *: $p < 0.05$, **: $p < 10^{-2}$, ***: $p < 10^{-3}$. Data and statistical tests for the non-switched animals reproduced from the main text.)

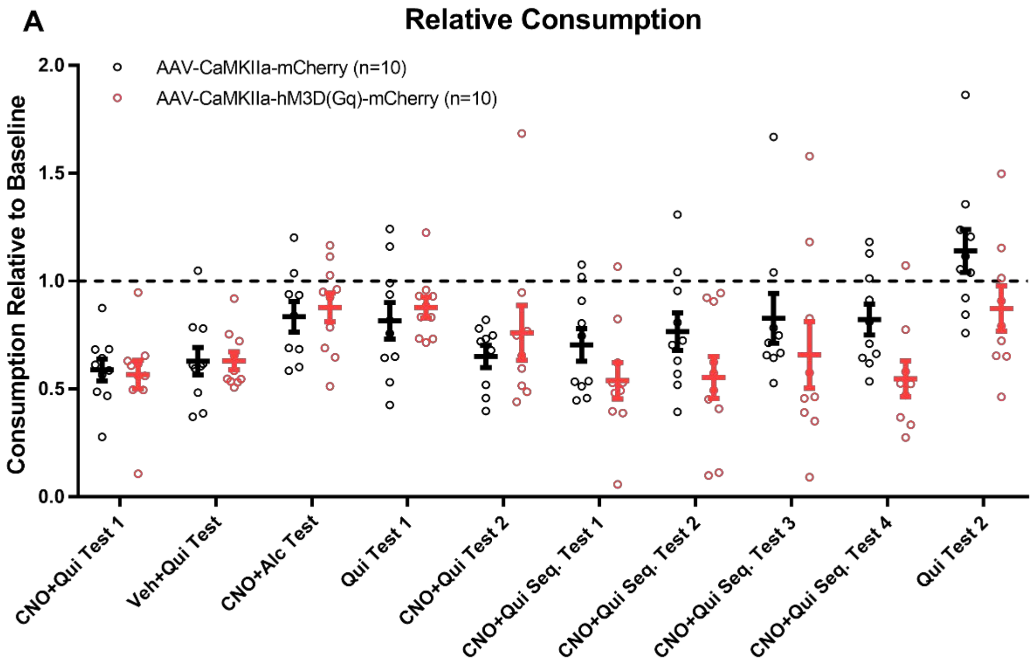

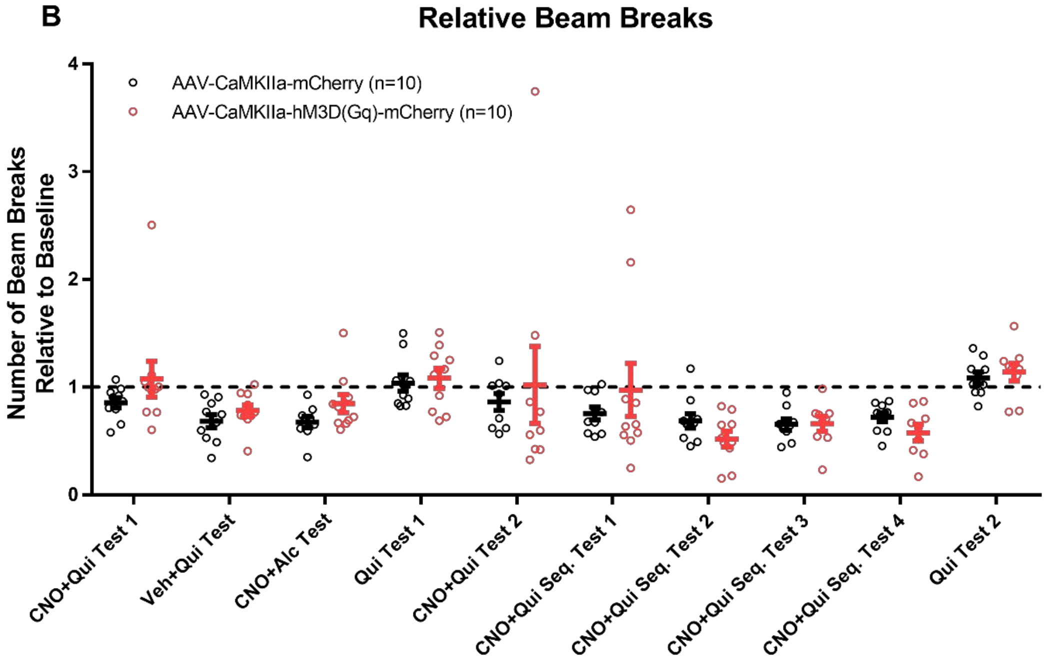

Supplemental Figure 15: Behavioral tests in DREADD and control P rats. Tests were performed sequentially from left to right with regular 2CAP session interspersed. All animals underwent a CNO+quinine test first. Half of the animals then underwent a vehicle+quinine test, a regular quinine test with no injection, then a CNO+alcohol test. The other animals underwent the same tests, but in reversed order. All animals then underwent a CNO+quinine test, followed by a sequence of four CNO+quinine tests for four days (one each day, no regular 2CAP sessions between). Finally, all animals underwent a quinine test with no injection. No differences were observed between number of beam breaks between

control and DREADD animals the two groups of tests discussed in the main text or as post-doc comparisons for individual tests. (Data are presented as mean +/- sem)

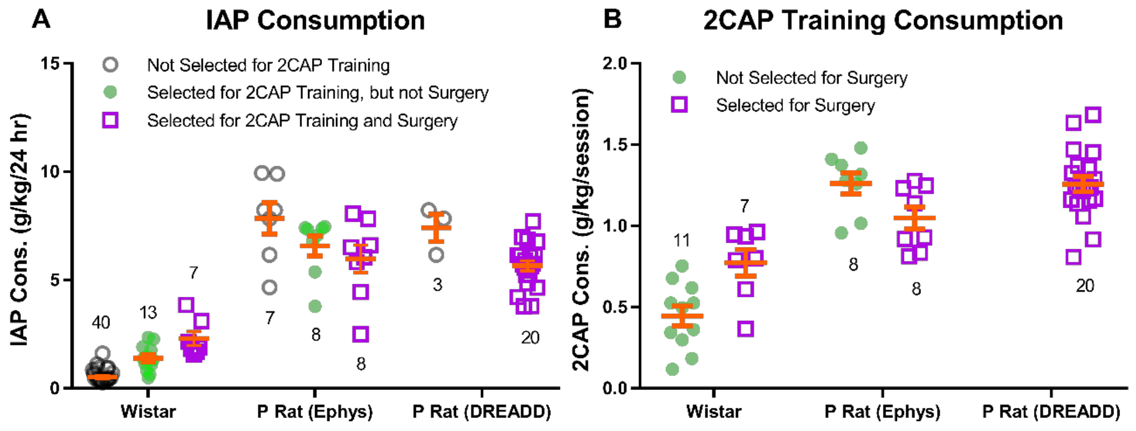

Supplemental Figure 16: Consumption results for animals during IAP and 2CAP training. Animals were primarily selected based on average consumption levels. However, this was not always possible due to the sizes of the cohorts. Also, some animals were not selected due to health concerns. In general, higher drinking Wistars and lower drinking P rats were selected. (Number of animals shown in figure. Data are presented as mean +/- sem.)

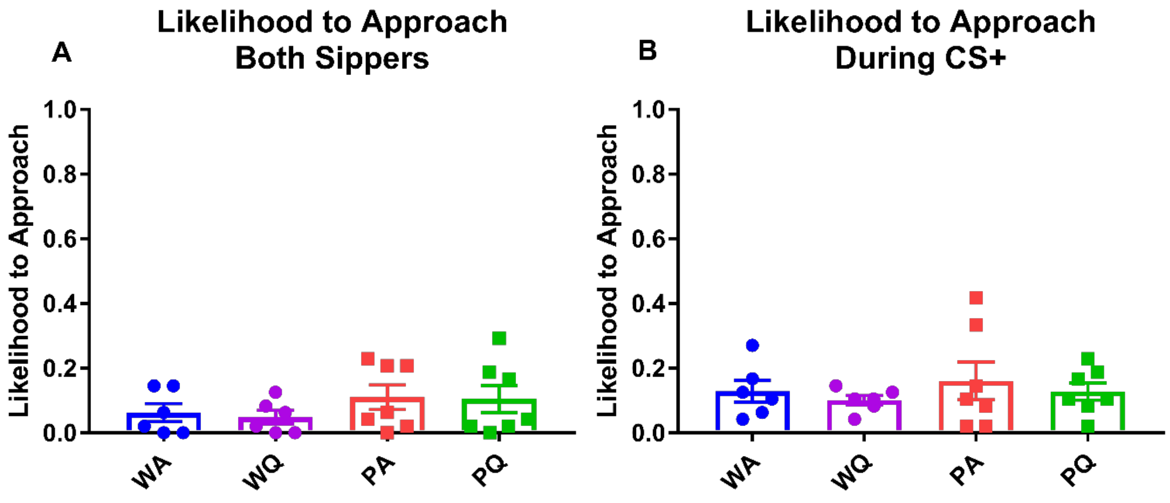

Supplemental Figure 17: Animals were unlikely to approach both sippers during access (i.e., correct from the incorrect sipper to the correct sipper) or approach the correct sipper during the CS+. No significant differences were observed between groups. (N=6 Wistars. N=7 P rats. Data are presented as mean +/- sem.)

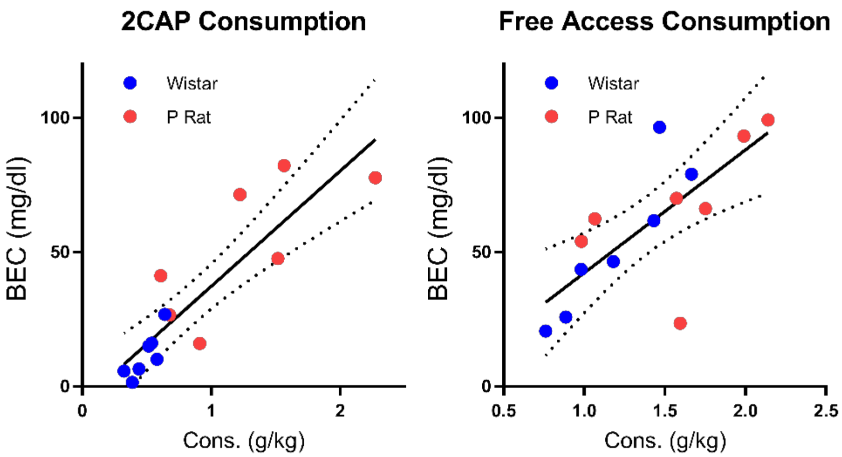

Supplemental Figure 18: Blood ethanol concentration measurements for regular 2CAP sessions and free access sessions. During the free access session, the animal was given unlimited access to both sippers for the same time period as a regular 2CAP session. The free access sessions and the regular 2CAP sessions shown here were not part of the rest of the analysis. A fit of the regular 2CAP intake vs. BEC for all animals found a significant slope (mean +/- SE: 42.94 +/- 6.87, $F(1,12) = 39.02$, $p < 0.0001$, $R^2 = 0.76$). Similarly, a fit of the free access intake vs. BEC for all animals also found a significant slope (mean +/- SE: 45.78 +/- 12.09, $F(1,12) = 14.34$, $p = 0.0026$, $R^2 = 0.54$). (N=7 Wistars. N=7 P rats.)

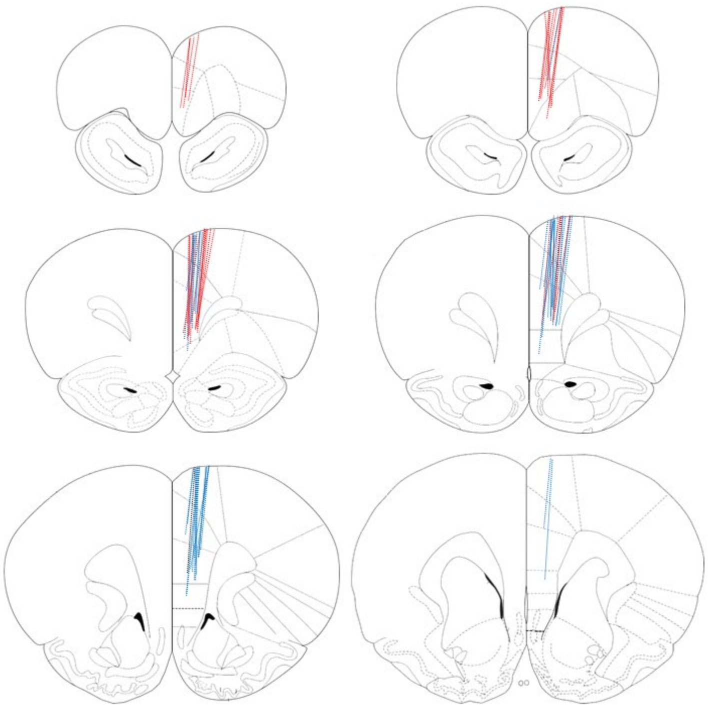

Supplemental Figure 19: Placement tracks for all Wistars (blue) and P rats (red). Note that these tracks represent the final location of the probes. Based on how frequently the probes were moved and the distance traveled, the initial quinine test recordings (which form the bulk of the analysis) occurred ~300-500 um above the final positions show in these tracks. Atlas image reproduced from [1].

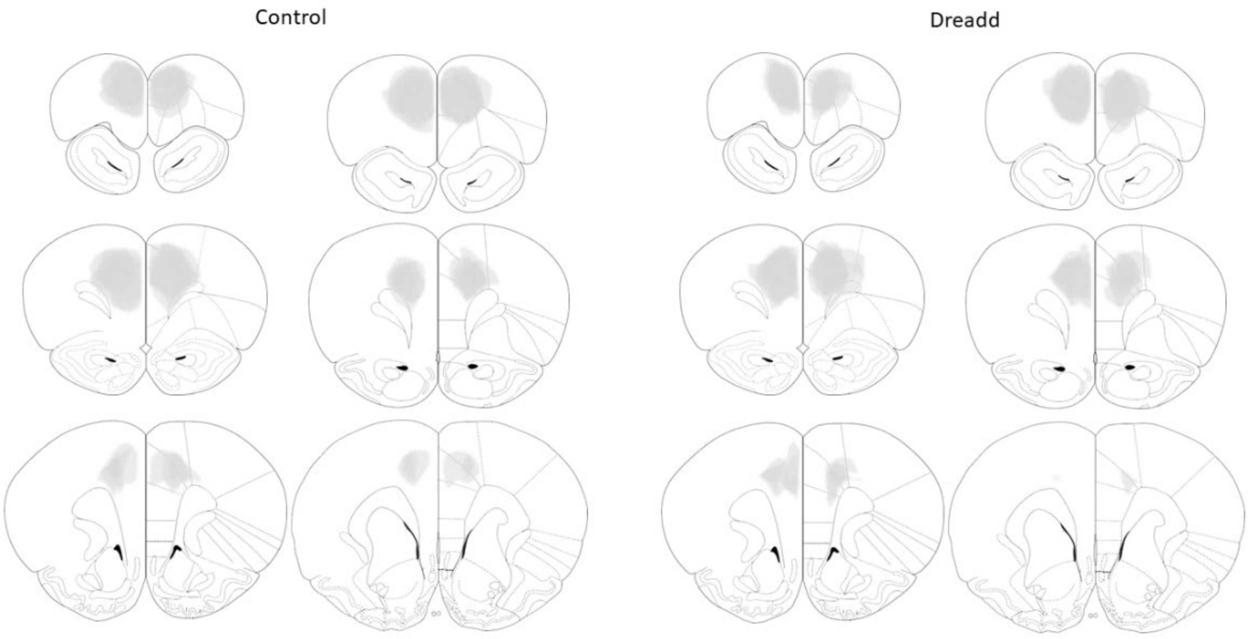

Supplemental Figure 20: Virus expression for control and DREADD animals. Expression was verified for all animals. Atlas image reproduced from [1].

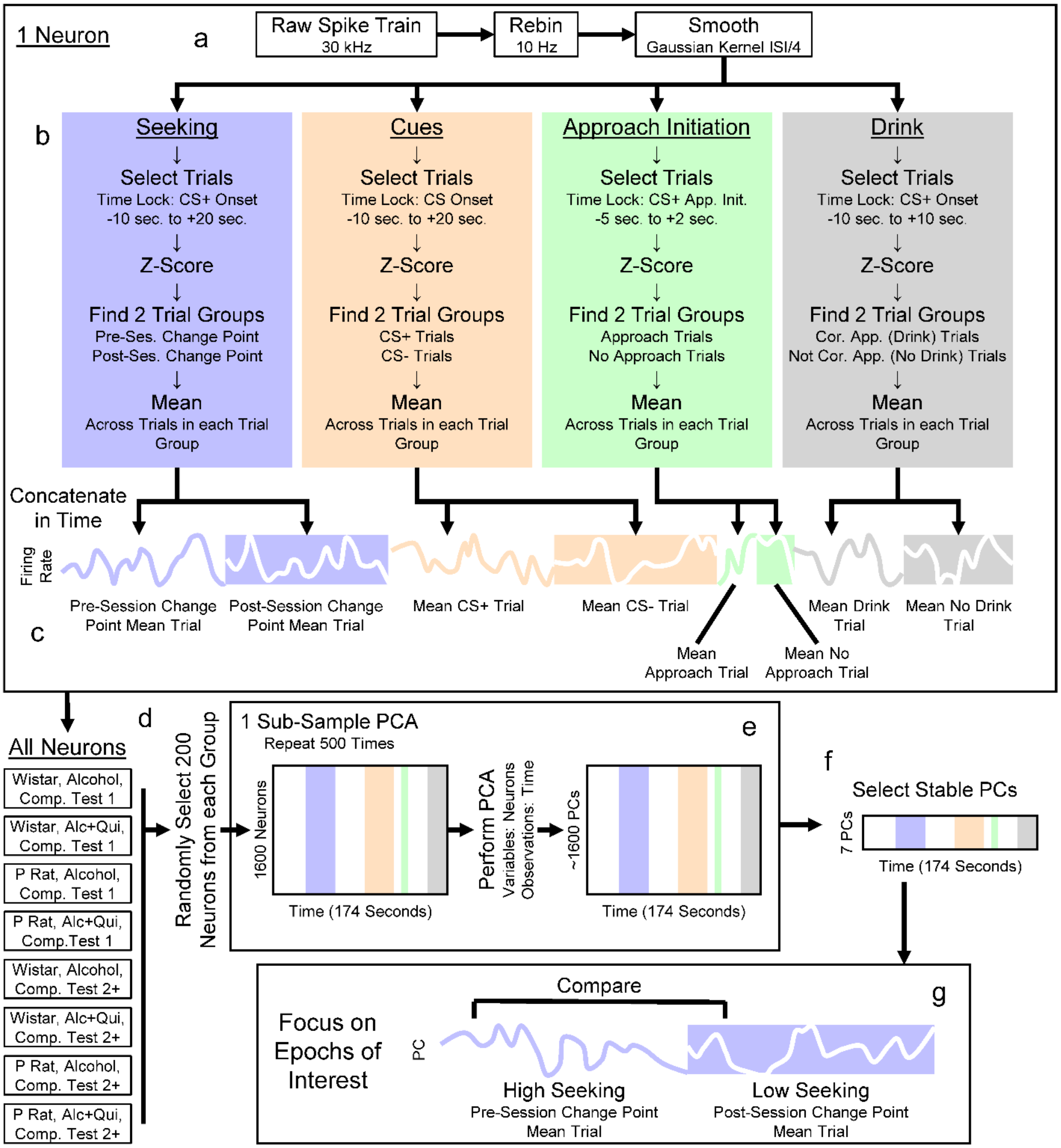

Supplemental Figure 21: PCA workflow. a) Raw data was recorded and spike sorted at 30 kHz, then downsampled to 10 Hz, then smoothed with a Gaussian kernel (standard deviation 25% of mean ISI separation for each neuron). b) For each segment of the decision-making process of interest, trials were selected based on a time lock point and a time window. The data were z-scored across all trials of interest. The trials were divided into two groups and means were taken across trials in each group. c) All mean trials of interest were concatenated for each neuron. d) Neurons were group by experimental conditions (8 groups: P rat vs. Wistar, alcohol vs. alcohol+quinine, Compulsion Test 1 vs. Compulsion Test 2+). e) Principal Component Analysis (PCA) was conducted using multiple subsamples to control for neuron yield in each experimental group. In each PCA iteration, 200 neurons from each experimental group were randomly selected. PCA was performed using neurons as variables and time as observations to assess

neural population firing patterns through trials of interest. f) Stable PCs were identified by comparing mean signal variance to variance across PCA iterations. g) Specific comparisons were made between PCs to assess encoding of various decision-making related variables, such as high seeking trials vs. low seeking trials.

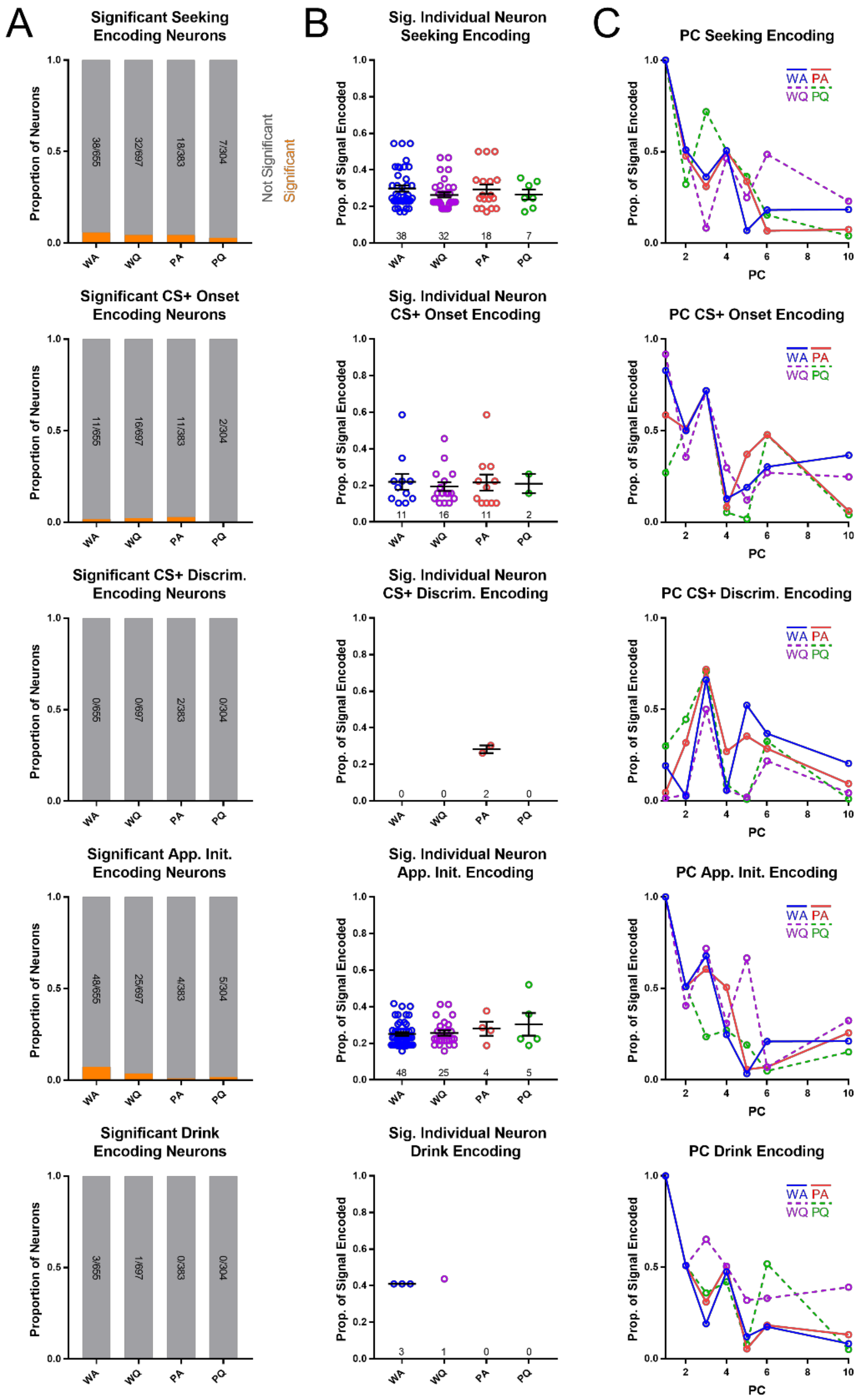

Supplemental Figure 22: Neural decoding/encoding analysis. We calculated the average neuron firing rate from the smoothed spike trains during the various epochs of interest on each trial. We then calculated the mutual information between the neuron firing rates and the variable of interest. For example, when assessing seeking encoding, we calculated the average firing rate during the -3 to -1 seconds before the CS+ onset and measured how well the firing rate predicted whether the trial was a seeking trial or a non-seeking trial. We binned the firing rates into two equal count states (high firing rate and low firing rate). We assessed significance by randomly permuting the firing rate states up to 100,000 times and measured the p-value as the share of randomized data mutual information values greater than or equal to the mutual information value from the original data. We then performed false discover rate control ($p < 0.05$) to correct for multiple comparisons. a) Very few neurons were found to significantly encode the decision-making variables of interest. b) The mutual information values for the significant encoders were relatively similar across strain and liquid groups. c) Next, we used a similar method to examine encoding by PCs. Because the PC values are derived from firing rates averaged over trials, it was not possible to directly assess encoding by PCs of decision-making relevant variables across trials. Therefore, we examined how well PCs encoded decision-making relevant variables across PCA subsampling trials (see Supplemental Figure 21e). Like the individual neuron analysis, we averaged the PC values (projected by strain and liquid type, as in the PCA presented in the manuscript) during the epochs of interest. We binned the PC values into two equal count states (high and low), calculated the mutual information with the variable of interest across the subsample trials, and measured significance by randomly permuting the variable of interest across subsample trials 1,000 times. We found most PCs encoded the variables of interest to a high degree, particularly PC 1, which frequently perfectly encoded the signal of interest. Note that all the mutual information values for all PCs was found to be highly significant, but because the number of data points was controlled by the number of subsampling trials, these significance measurements were not meaningful. (Number of neurons shown in the figure. In b, data are presented as mean +/- sem.)

References

1. Paxinos, G. & Watson, C. *The Rat Brain* (Academic Press, Ltd., San Diego, CA, 1998).
