# Supplementary material for: Compulsive alcohol drinking in rodents is associated with altered representations of behavioral control and seeking in dorsal medial prefrontal cortex
Source: Nat Commun. 2022 Jul 9;13:3990. doi: 10.1038/s41467-022-31731-4 (PMC9271071; doi:10.1038/s41467-022-31731-4)
Supplement: Supplementary file 3 — Reporting Summary [file 41467_2022_31731_MOESM3_ESM.pdf]

nature research

Corresponding author(s): Timme, Nicholas

Last updated by author(s): Apr 25, 2022

Reporting Summary

Nature Research wishes to improve the reproducibility of the work that we publish. This form provides structure for consistency and transparency in reporting. For further information on Nature Research policies, see our Editorial Policies and the Editorial Policy Checklist.

Statistics

For all statistical analyses, confirm that the following items are present in the figure legend, table legend, main text, or Methods section.

n/a	Confirmed	
☐	☒	The exact sample size (n) for each experimental group/condition, given as a discrete number and unit of measurement
☐	☒	A statement on whether measurements were taken from distinct samples or whether the same sample was measured repeatedly
☐	☒	The statistical test(s) used AND whether they are one- or two-sided<br>*Only common tests should be described solely by name; describe more complex techniques in the Methods section.*
☐	☒	A description of all covariates tested
☐	☒	A description of any assumptions or corrections, such as tests of normality and adjustment for multiple comparisons
☐	☒	A full description of the statistical parameters including central tendency (e.g. means) or other basic estimates (e.g. regression coefficient) AND variation (e.g. standard deviation) or associated estimates of uncertainty (e.g. confidence intervals)
☐	☒	For null hypothesis testing, the test statistic (e.g. F, t, r) with confidence intervals, effect sizes, degrees of freedom and P value noted<br>*Give P values as exact values whenever suitable.*
☒	☐	For Bayesian analysis, information on the choice of priors and Markov chain Monte Carlo settings
☐	☒	For hierarchical and complex designs, identification of the appropriate level for tests and full reporting of outcomes
☐	☒	Estimates of effect sizes (e.g. Cohen's d, Pearson's r), indicating how they were calculated

Our web collection on statistics for biologists contains articles on many of the points above.

Software and code

Policy information about availability of computer code

Data collection	MedPC (Version 4.1), OpenEphys (Version 0.4.0), Logitech Webcam Software (Version 2.80.853.0)
Data analysis	Kilosort (Version 2), DeepLabCut (Version 2), Matlab (Version 2020a), Custom Matlab Code (included in figshare repository, https://doi.org/10.6084/m9.figshare.19387511.v1), GraphPad Prism (Version 7)

For manuscripts utilizing custom algorithms or software that are central to the research but not yet described in published literature, software must be made available to editors and reviewers. We strongly encourage code deposition in a community repository (e.g. GitHub). See the Nature Research guidelines for submitting code & software for further information.

Data

Policy information about availability of data

All manuscripts must include a data availability statement. This statement should provide the following information, where applicable:

- Accession codes, unique identifiers, or web links for publicly available datasets
- A list of figures that have associated raw data
- A description of any restrictions on data availability

All spike sorted data, behavioral data, and analysis software are available in a figshare repository (https://doi.org/10.6084/m9.figshare.19387511.v1).

Field-specific reporting

Life sciences study design

All studies must disclose on these points even when the disclosure is negative.

Sample size	Sample size impacted this study in two key ways: via animals and neurons. We did not perform a sample-size calculation prior to gathering data. Such calculations are difficult to perform for neurons in this case given the population signal analysis of the individual neuron firing patterns. Instead, we sought to obtain hundreds of neurons from each experimental group (which we achieved). Furthermore, we sought to record these neurons from at least 5 animals in each experimental group (which we achieved) to insure that results were not attributable to an anomalous animal. To verify this, we conducted a leave one out analysis where we reran the entire analysis after removing an animal (see manuscript for details). These quantities of animals and neurons have traditionally been used in this field because they supply sufficient confidence that moderate effect size phenomena can be observed among neurons and that results are not due to an anomalous animal.
Data exclusions	Data was only excluded in certain limited circumstances and these exclusions are described in the manuscript. Instances of excluded data include animals that did not survive electrode placement surgery, animals whose electrode implants failed, animals that did not drink alcohol in their home cage, animals that could not learn the task, and fluid measurements that were in error due to leaks. Criteria for exclusion were not established prior to the study.
Replication	To the best of our knowledge, no one has attempted to replicate this study due to the laborious and time consuming nature of the study.
Randomization	Due to timing restrictions, subjects were not randomized across strain. It was only feasible to maintain a single cohort of 4 animals of the same strain at a time, so the experiment utilized 1 cohort of 4 P rats, 1 cohort of Wistars, 1 cohort of P rats, and 1 cohort of Wistars, in that order.
Blinding	Due to timing restrictions, all animals in a cohort were the same strain and had preferred drinking times during the day. So, it was not possible to blind experimenters to the identify of the animals.

Reporting for specific materials, systems and methods

We require information from authors about some types of materials, experimental systems and methods used in many studies. Here, indicate whether each material, system or method listed is relevant to your study. If you are not sure if a list item applies to your research, read the appropriate section before selecting a response.

Materials & experimental systems

n/a	Involved in the study
☒	☐ Antibodies
☒	☐ Eukaryotic cell lines
☒	☐ Palaeontology and archaeology
☐	☒ Animals and other organisms
☒	☐ Human research participants
☒	☐ Clinical data
☒	☐ Dual use research of concern

Methods

n/a	Involved in the study
☒	☐ ChIP-seq
☒	☐ Flow cytometry
☒	☐ MRI-based neuroimaging

Animals and other organisms

Policy information about studies involving animals; ARRIVE guidelines recommended for reporting animal research

Laboratory animals	Rattus norvegicus, Wistars and Alcohol Preferring P Rats, male, 12-24 weeks
Wild animals	This study did not involve wild animals.
Field-collected samples	This study did not involve animals collected from the field.
Ethics oversight	Indiana University - Purdue University Indianapolis School of Science Institutional Animal Care and Use Committee

Note that full information on the approval of the study protocol must also be provided in the manuscript.
